# Evaluation of Bread Crumbs as a Potential Carbon Source for the Growth of Thraustochytrid Species for Oil and Omega-3 Production

**DOI:** 10.3390/nu6052104

**Published:** 2014-05-23

**Authors:** Tamilselvi Thyagarajan, Munish Puri, Jitraporn Vongsvivut, Colin J. Barrow

**Affiliations:** Centre for Chemistry and Biotechnology, Deakin University, Pigdons Road, Waurn Ponds, Victoria 3217, Australia; E-Mails: tthyagar@deakin.edu.au (T.T.); munish.puri@deakin.edu.au (M.P.); p.vongsvivut@deakin.edu.au (J.V.)

**Keywords:** thraustochytrids, biodiesel, feed supplement, static fermentation

## Abstract

The utilization of food waste by microorganisms to produce omega-3 fatty acids or biofuel is a potentially low cost method with positive environmental benefits. In the present study, the marine microorganisms *Thraustochytrium* sp. AH-2 and *Schizochytrium* sp. SR21 were used to evaluate the potential of breadcrumbs as an alternate carbon source for the production of lipids under static fermentation conditions. For the *Thraustochytrium* sp. AH-2, submerged liquid fermentation with 3% glucose produced 4.3 g/L of biomass and 44.16 mg/g of saturated fatty acids after seven days. Static fermentation with 0.5% and 1% breadcrumbs resulted in 2.5 and 4.7 g/L of biomass, and 42.4 and 33.6 mg/g of saturated fatty acids, respectively. Scanning electron microscopic (SEM) studies confirmed the growth of both strains on breadcrumbs. Attenuated total reflection Fourier transform infrared (ATR-FTIR) spectroscopy for both strains were consistent with the utilization of breadcrumbs for the production of unsaturated lipids, albeit at relatively low levels. The total lipid yield for static fermentation with bread crumbs was marginally lower than that of fermentation with glucose media, while the yield of unsaturated fatty acids was considerably lower, indicating that static fermentation may be more appropriate for the production of biodiesel than for the production of omega-3 rich oils in these strains.

## 1. Introduction

Thraustochytrids are marine protists that belong to Labyrinthulomycetes and were first reported by Sparrow in 1936. These microorganisms are epibiotic in nature and represent a diverse group of organisms living in marine and estuarine habitats throughout the world and exhibiting a saprotrophic mode of nutrition [1]. Due to their ability to produce a large amount of oil, including polyunsaturated fatty acids (PUFAs), research has focused on lowering their cost of production using low cost carbon and nitrogen sources, particularly for large-scale industrial fermentation [2]. Biomass produced through heterotrophic fermentation of thraustochytrids is a potentially sustainable approach to the production of PUFAs for food, feed and supplement applications and oil for biofuel applications [3].

Thraustochytrids can act as microbial cell factories for the production of omega-3 PUFAs, squalene, and other secondary metabolites such as carotenoids and sterols, along with enzymes and extracellular polysaccharides [2]. For commercial utility in the production of these materials, thraustochytrid growth should be low cost, particularly to compete in the food market as a replacement for fish oils. Because heterotrophic organisms require a carbon source for bioconversion into oil, they are more expensive to grow in terms of consumables than autotrophic organisms. Heterotrophic fermentation has some advantages over autotrophic fermentation, particularly the ability to use standard industrial fermentation equipment at scale and to get much higher cell density than can be achieved with autotrophic organisms, which often need complex and expensive equipment to enable light to reach the cells while preventing contamination [4,5]. The cost of glucose as a carbon source in the growth medium to produce biomass can account for up to 30% of the overall production cost and so commercial biofuel production using heterotrophic fermentation requires the use of low cost carbon sources such as glycerol or food waste [6,7]. Food wastes generated worldwide are about 1.3 billion tons [8]. Management of food wastes through landfill dumping is common and is environmentally problematic. Food in landfills can rot and release methane gas, which is a major contributor to carbon emissions worldwide [9].

Research on using microbial fermentation to convert food waste into value added products is limited [10]. *Schizochytrium mangrovei* and *Chlorella pyrenoidosa* have been grown using food waste obtained by fungal hydrolysis and were reported to produce biomass potentially useful as a feed supplement or for biodiesel production [6]. *Schizochytrium mangrovei* KF6 was also reported to utilize processed bread crust to produce docosahexaenoic acid (DHA) from shake flask fermentation at 200 rpm under fluorescent light for eight days [11]. Polyunsaturated fatty acids were produced by *Mortierella alpina* utilizing rice bran as the carbon source in a solid-state column reactor under static conditions. Static conditions, where there is no agitation during the growth phase, are often used in solid substrate fermentation, as opposed to submerged liquid fermentation which requires higher energy inputs for continuous shaking of the flask at constant speed [12]. Other inexpensive carbon sources derived from food wastes that were studied includes okara powder [13], residues from beer and potato processing [14], sweet sorghum juice [15], coconut water [16], marine aquaculture waste water [17] and crude glycerol [18].

The objective of the present study was to investigate the ability of some thraustochytrid strains to utilize bakery waste, specifically breadcrumbs (BC), as an alternate carbon source in the fermentation media to produce lipids under static conditions. Scanning electron microscopy (SEM) was used to investigate the growth pattern of these microorganisms during static fermentation, and attenuated total reflection Fourier transform infrared (ATR-FTIR) spectroscopic analysis was performed to observe the production of unsaturated fatty acids during fermentation. ATR-FTIR spectroscopy and SEM were shown to be useful for monitoring static fermentation.

## 2. Experimental Section

All chemicals including fatty acid methyl ester standards used in this study were procured from Sigma-Aldrich (Sydney, Australia) and Merck Chemicals (Victoria, Australia) and were of analytical grade.

### 2.1. Preparation of Seed Culture

Thraustochytrid strains used in the study were *Thraustochytrium* sp. AH-2 (ATCC^®^ PRA-296™, Manassas, VA, USA) and *Schizochytrium* sp. SR21 (ATCC^®^ MYA-1381™, Manassas, VA, USA) and these strains were procured from the American Type Culture Collection (ATCC, Manassas, VA, USA), and grown in liquid media containing 1 g yeast extract, 15 g peptone, 20 g glucose (1 g yeast extract, 1 g peptone, 5 g glucose for *Schizochytrium* sp. SR21) in 1 L of artificial sea water (ASW) at 70% strength, according to ATCC product information sheets. In brief, the cultures were grown at 20 °C with shaking speed of 120 rpm for 96 h. Seed culture of 5% (v/v) culture was used in subsequent submerged liquid glucose fermentation and static fermentation with breadcrumbs as an alternate carbon source.

### 2.2. Submerged Liquid Fermentation with Glucose Medium

Cultures were grown in 250 mL flasks with 50 mL of medium altered and adopted from Li *et al.* [19]. Flasks were kept at 20 °C with shaking speed of 120 rpm for 7 days with medium at a starting pH of 6.0 (prior to autoclaving) and the pH was not controlled over the fermentation period. Flasks were collected for the dry weight determination and fatty acid estimation, each for 24 h.

### 2.3. Preparation of Bakery Waste Bread Crumbs (BC) for Static Fermentation

Breadcrumbs (crude powder; non-uniform size) were purchased from a local bakery (Geelong, Australia) for evaluating their utilization under static fermentation. BC powder was used for carrying fermentation experiments. Static fermentation was carried with the same media as submerged fermentation but with BC (at 0.5% and 1%) substituted for glucose in the media. Flasks were kept under static conditions in an incubator at 20 °C for 7 days, with pH of medium adjusted at 6.0 prior to autoclaving but not adjusted during the fermentation phase. Flasks were inoculated with 5% (v/v) seed culture (Section 2.1) under aseptic conditions. Samples were collected for the cell dry weight and fatty acid estimation after 24 h. Freeze-dried BC were analyzed using an EuroEA elemental analyzer (Euro Vector, Milan, Italy) to determine the percentages of carbon and nitrogen content.

### 2.4. Scanning Electron Microscopy (SEM)

A small flake of freeze-dried cells was mounted onto carbon tape on an aluminum stub and air dried, after which 60 nm of gold was deposited on its surface using a sputter coater. The cells were examined under a scanning electron microscope (SEM Supra 55 VP, Zeiss, Berlin, Germany) at accelerating voltage 3–5 KV using secondary electron detector.

### 2.5. Fatty Acid Extraction and Gas Chromatography (GC) Analysis

Fatty acid extraction was performed as previously described with some modifications [20]. In brief, 10 mg of freeze-dried cells were used for lipid extraction. Fatty acids were extracted with a mixture containing a 2:1 ratio of chloroform to methanol and repeated 3 times. For trans-esterification, 1 mL toluene was added followed by addition of 200 µL of internal standard, methyl nonadecanoate (C19:0) and 200 µL of butylated hydroxytoluene (BHT). Acidic methanol (2 mL) was also added to the tube and kept for overnight incubation at 50 °C. Fatty acid methyl esters (FAMEs) were extracted into hexane. The hexane layer was removed and dried over sodium sulphate. FAMEs were concentrated using nitrogen gas prior to GC analysis [21]. The samples were analyzed using a GC-FID system (Agilent Technologies, 6890N, Santa Clara, CA, USA). The GC instrument was equipped with a capillary column (Suplecowax 10, 30 × 0.25 mm, 0.25 µm thickness). Helium was used as the carrier gas at a flow rate of 1.5 mL·min^−1^. The injector was maintained at 250 °C and a sample volume of 1 µL was injected. Fatty acids were identified by comparison to external standards (Sigma-Aldrich, Sydney, Australia). Peaks were quantified with Chemstation chromatography software (Agilent Technologies, Santa Clara, CA, USA) and corrected using theoretical relative FID response factors [22]. Samples are analyzed in duplicate and compared to external standards.

### 2.6. Attenuated Total Reflection Fourier Transform Infrared (ATR-FTIR) Spectroscopic Analysis

ATR-FTIR measurements of the freeze-dried samples were conducted using an Alpha FTIR spectrometer (Bruker Optik GmbH, Ettlingen, Germany) equipped with a deuterated triglycine sulfate (DTGS) detector and a single-reflection diamond ATR sampling module (Platinum ATR QuickSnap™, Ettlingen, Germany). The ATR-FTIR spectra were acquired at 4-cm^−1^ spectral resolution with 64 co-added scans within the 4000–400 cm^−1^ spectral region. Blackman-Harris 3-Term apodization, power-spectrum phase correction, and zero-filling factor of 2 were set as default acquisition parameters using OPUS 6.0 software suite (Bruker Optik GmbH, Ettlingen, Germany). Background spectra of a clean ATR surface were acquired prior to each sample measurement using the same acquisition parameters.

### 2.7. Statistical Analysis

Data were statistically compared using one-way analysis of variance (Anova), and the significant difference was identified using Tukey’s and Scheffe tests. The analysis was carried out using SPSS software (IBM^®^ SPSS^®^ Statistics 20, Sydney, Australia).

## 3. Results and Discussion

*Thraustochytrium* sp. AH-2 was previously isolated from coastal and mangrove habitats of Goa and further studied for its extracellular alkaline lipase production [23]. In the present study, control fermentation profiles were obtained using submerged liquid fermentation with 3% glucose as the carbon source. Under glucose conditions, biomass of 4.3 g/L and total lipid yield of 941 mg/L were achieved (Table 1). The biomass and lipid yield for our species is similar to that reported for *T. aureum* ATCC 34304 [24], although optimization of controlled fermentation conditions would probably enable higher biomass and lipid production for strain AH-2. Oleic acid (C18:1n9) was the major fatty acid at 63.19 mg/g, followed by palmitic acid (C16:0) 32.33 mg/g, DHA (C22:6n3) 23.74 mg/g and stearic acid (C18:0) 11.82 mg/g. C14:0, C15:0. C16:1n7, C17:0, C17:1n7 were present in lower amounts. DHA (C22:6n3) was the major PUFA, followed by docosapentaenoic acid (DPA) (C22:5n6) at 4.32 mg/g and eicosapentaenoic acid (EPA) (C20:5n3) at 3.03 mg/g.

**Table 1:** Fermentation profiles for *Thraustochytrium* sp. AH-2.

Fermentation type	Biomass (mg/L)	Lipids (mg/L)	Saturated fatty acids ^1^ (mg/g)	Mono-unsaturated fatty acids ^2^ (mg/g)
Submerged liquid fermentation (3% glucose)	4300	941.32	44.16	63.19
Static fermentation (0.5% bread crumbs)	2530	260.0	42.4	29.00
Static fermentation (1% bread crumbs)	4760	390.0	33.6	22.6

^1^ Palmitic acid (C16:0); stearic acid (C18:0); ^2^ Oleic acid (C18:1n9).

### 3.1. Fermentation Growth Using Bread Crumbs as the Carbon Source

Elemental analysis of the freeze-dried BC revealed approximately 40.95% carbon and 3.23% nitrogen. The fatty acid analysis of unfermented BC is presented in Table 2 and shows oleic acid as the major fatty acid in the profile, followed by palmitic acid (C16:0), stearic acid (C18:0), linoleic acid (18:2n6) and other fatty acids were also present. The polyunsaturated fatty acids EPA, DPA and DHA were not detected in the fatty acid profile of BC.

**Figure 1:**
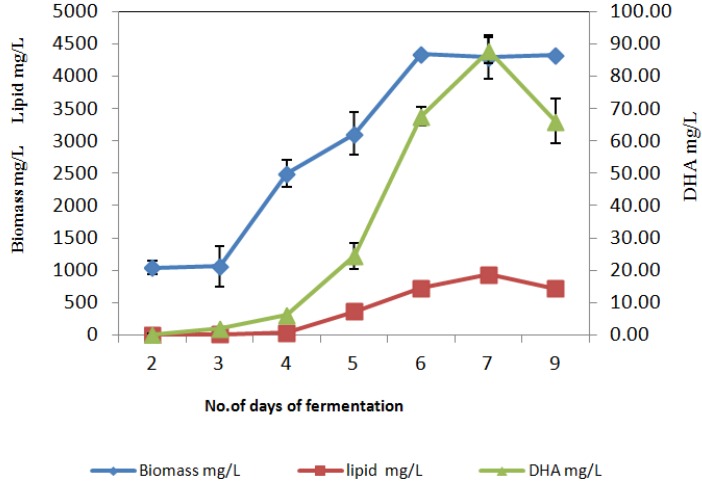
Fermentation profile of *Thraustochytrium* sp. AH-2 under submerged liquid fermentation with 3% glucose as the carbon source.

*Thraustochytrium* sp. AH-2 was grown using BC as the carbon source and the results compared with those obtained using glucose. BC is known to contain primarily complex carbohydrate in the form of starch [25]. BC was used at levels of 0.5% and 1%. Higher levels of 3% and 5% BC gave no improvement in cell growth and made lipid extraction difficult and were not pursued further. Fermentation with 0.5% BC gave 2.5 g/L of biomass and 260 mg/L of total lipid yield. Fermentation with 1% BC gave 4.7 g/L of biomass and 390 mg/L of total lipid yield (Figure 1). Compared to liquid fermentation with glucose, the total lipid yield was relatively low for static fermentation. 

**Table 2:** Fatty acid profile ^1^ (mg/g) of unfermented bread crumbs, submerged liquid fermentation and static fermentation with bread crumbs, for *Thraustochytrium* sp. AH-2.

C16:0	C18:0	C18:1n9	C18:2n6	C18:3n3	C20:5n3	C22:5n3	C22:6n3	others
*Unfermented bread crumbs fatty acid profile*								
2.20	1.50	3.10	1.00	0.00	0.00	0.00	0.00	1.50
*Submerged liquid fermentation fatty acid profile* ^2^								
32.33 ^a^	11.82 ^a^	63.19 ^a^	2.76 ^a^	0.00 ^a^	3.03	4.32	23.74	33.35
*Static fermentation fatty acid profile* ^3^								
25.9 ^b^^,4^	16.5 ^b^	29.0 ^b^	12.9 ^b^	1.2 ^b^	0.00	0.57	2.40	14.40
20.4 ^b^^,5^	13.2 ^b^	22.6 ^b^	11.2 ^b^	1.2 ^b^	0.00	0.00	1.30	11.50

^1^ Palmitic acid (C16:0); stearic acid (C18:0); oleic acid (C18:1n9); linoleic acid (C18:2n6); linolenic (C18:3n3); EPA (C20:5n3); DPA (C22:5n3); DHA (C22:6n3); ^2^ Submerged liquid fermentation media with 3% glucose incubated for 7 days at 20 °C with shaking speed of 120 rpm; ^3^ Static fermentation with same medium composition as submerged liquid fermentation, with breadcrumbs as 5 and 10 g substituted for glucose in 1litre of 70% ASW at pH 6; ^4^ Static fermentation with 0.5% BC, incubated at 20 °C for 7 days; ^5^ Static fermentation with 1% BC, incubated at 20 °C for 5 days; ^a,b^ indicates statistically significant difference for submerged liquid fermentation (^a^) and static fermentation (^b^) (*p* < 0.05).

Under static fermentation, unsaturated fatty acids were poorly synthesized, although the presence of C18:3n3 (linolenic acid), EPA and DHA were clearly observed. Previous study on *Schizochytrium mangrovei* KF6 under heterotrophic conditions with processed bread crust also reported low levels of unsaturated fatty acids [11]. In general, monosaccharide sugars in the media result in the production of higher levels of PUFAs compared to di- and poly-saccharides [26]. As BC contains mainly starch, the observed poor synthesis of unsaturated fatty acids is not unexpected. In this study, the maximum fatty acid profile with 1% BC was observed on day 5, after which fatty acid content decreased, probably due to lipid consumption by the organism. Lipids and fatty acids accumulated in oleaginous microorganisms can act as energy sources for growth that are utilized when there is a lack of available carbon in the media [27].

The total amount of saturated fatty acids, which was primarily C16:0 and C18:0, were 42.4 mg/g with 0.5% BC and 33.6 mg/g with 1% BC, whilst submerged liquid fermentation with glucose as the carbon source gave an only slightly higher level of saturated fatty acids at 44.1 mg/g. Since PUFA production is low and unsaturated production is relatively high, fermentation with BC provides a fatty acid profile more consistent with that of biofuel, than did submerged liquid fermentation. Static fermentation may be a useful method for converting BC to oil, since parameters are readily standardized at industrial scale.

### 3.2. Scanning Electron Microscopy (SEM) Observation of Cell Growth

**Figure 2:**
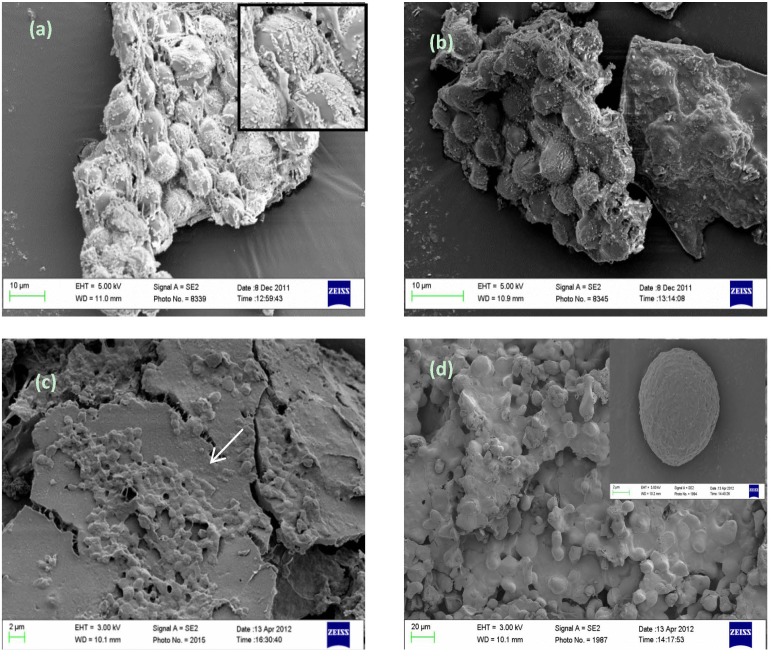
SEM images of freeze-dried cells for (**a**) freeze-dried cells of *Schizochytrium* sp. SR21 grown under submerged liquid fermentation; (**b**) *Schizochytrium* sp. SR21 cells grown with 1% BC as alternate carbon source; (**c**) *Thraustochytrium* sp. AH-2 cells grown with 1% BC as alternate carbon source; (**d**) *Thraustochytrium* sp. AH-2 grown under submerged liquid fermentation.

The fermentation growth for *Thraustochytrium* sp. AH-2 and *Schizochytrium* sp. SR21 were compared for BC and glucose as the carbon source using SEM. The morphology of freeze-dried unfermented BC was observed using SEM as a control material. The SEM images of cells grown using submerged liquid fermentation with glucose show spherical cells that are clumped together (Figure 2a). When grown in the presence of BC, cell clusters are attached to the BCs, confirming that cells do grow on this complex carbon source (Figure 2b,c).

### 3.3. ATR-FTIR Spectroscopy Analysis of Cell Lipid Content

**Figure 3:**
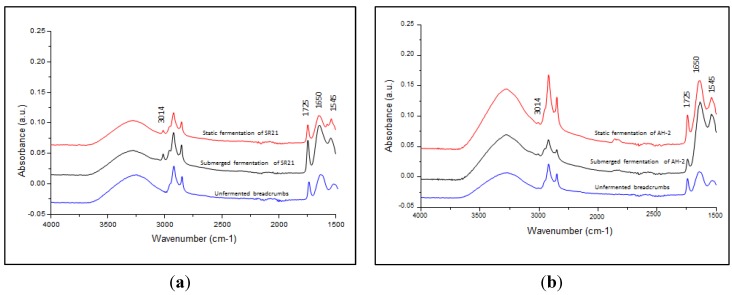
ATR-FTIR spectra of: (**a**) *Thraustochytrium* sp. AH-2; and (**b**) *Schizochytrium* sp. SR21. Black line—submerged fermentation; Blue line—unfermented breadcrumbs; Red line—static fermentation with breadcrumbs.

ATR-FTIR spectroscopic measurement of *Schizochytrium* sp. SR21 was performed to confirm the production of unsaturated fatty acids when BC was used as an alternative carbon source, in comparison to the submerged liquid fermentation with glucose of the same strain. Figure 3 shows the comparison of the ATR-FTIR spectral features of the raw unfermented BC, the static fermented 1% BC and glucose fermented cells. In particular, the olefinic C=CH stretching vibration found at ~3014 cm^−1^ is commonly known as a representative band for unsaturated fatty acids [28,29]. This band is clearly observed in the freeze-dried cells that were grown in 1% concentration of BC under static fermentation and with glucose, suggesting that observable amounts of unsaturated fatty acids were produced in the cells grown by both fermentation methods. The triplet bands found in the range of 3000–2800 cm^−1^, on the other hand, are attributed to C-H stretches of lipids and proteins [29]. At the low wavenumber region, the strong bands centered at 1650 and 1545 cm^−1^, known as amide I and II bands, respectively, occurred due to the protein moieties in the BC and the cells. The sharp band at 1725 cm^−1^, on the other hand, represents ν(C=O) stretches of ester functional groups from lipids and fatty acids, and is therefore indicative of total lipids produced by the cells [28,29,30]. According to the intensities of this band, fermentation with glucose led to a substantially higher amount of total lipids produced in the microorganisms. However, the ratios of unsaturated fatty acids per total lipids (*i.e.*, *I*_3014_/*I*_1725_) were found to be comparable between both fermentation approaches, suggesting that similar yields of unsaturated fatty acid can be achieved using BC as a carbon source under static fermentation of *Schizochytrium* sp. SR21. Therefore, growth of these strains on BC is potentially useful both for the utilization of food waste and the production of lipid.

## 4. Conclusions

*Thraustochytrium* sp. AH-2 and *Schizochytrium* sp. SR21 were tested for their ability to utilize BC during static fermentation as a low-cost and environmental friendly carbon source for producing oil for either biofuel (saturated fatty acid rich) or food (PUFA rich). The fatty acid profiles from *Thraustochytrium* sp. AH-2 indicated low levels of PUFA and higher levels of saturated oil. ATR-FTIR spectroscopy of *Schizochytrium* sp. SR21 was also consistent with higher levels of saturated fatty acids. Fermentation on BC containing complex carbohydrate appears to be more appropriate to the production of biofuel from these organisms than for the production of high levels of PUFA for food applications, due to the suppression of PUFA when BC is used as the carbon source.
